# Induction without methanol: novel regulated promoters enable high-level expression in *Pichia pastoris*

**DOI:** 10.1186/1475-2859-12-5

**Published:** 2013-01-24

**Authors:** Roland Prielhofer, Michael Maurer, Joachim Klein, Jana Wenger, Christoph Kiziak, Brigitte Gasser, Diethard Mattanovich

**Affiliations:** 1 University of Natural Resources and Life Sciences, Department of Biotechnology, Muthgasse 18, Vienna, 1190, Austria; 2 Austrian Centre of Industrial Biotechnology (ACIB GmbH), Muthgasse 11, Vienna, 1190, Austria; 3 University of Applied Sciences FH-Campus Vienna, School of Bioengineering, Muthgasse 18, Vienna, 1190, Austria; 4 Lonza AG, Rottenstraße 6, Visp, 3930, Switzerland

**Keywords:** *Pichia pastoris*, Heterologous protein production, Glucose-limited fed batch cultivation, Inducible promoter, High-affinity glucose transporter

## Abstract

**Background:**

Inducible high-level expression is favoured for recombinant protein production in *Pichia pastoris*. Therefore, novel regulated promoters are desired, ideally repressing heterologous gene expression during initial growth and enabling it in the production phase. In a typical large scale fed-batch culture repression is desired during the batch phase where cells grow on a surplus of e.g. glycerol, while heterologous gene expression should be active in the feed phase under carbon (e.g. glucose) limitation.

**Results:**

DNA microarray analysis of *P. pastoris* wild type cells growing in glycerol-based batch and glucose-based fed batch was used for the identification of genes with both, strong repression on glycerol and high-level expression in the feed phase. Six novel glucose-limit inducible promoters were successfully applied to express the intracellular reporter eGFP. The highest expression levels together with strong repression in pre-culture were achieved with the novel promoters P_G1_ and P_G6_.

Human serum albumin (HSA) was used to characterize the promoters with an industrially relevant secreted protein. A P_G1_ clone with two gene copies reached about 230% of the biomass specific HSA titer in glucose-based fed batch fermentation compared to a P_GAP_ clone with identical gene copy number, while P_G6_ only achieved 39%. Two clones each carrying eleven gene copies, expressing HSA under control of P_G1_ and P_G6_ respectively were generated by post-transformational vector amplification. They produced about 1.0 and 0.7 g L^-1^ HSA respectively in equal fed batch processes. The suitability in production processes was also verified with HyHEL antibody Fab fragment for P_G1_ and with porcine carboxypeptidase B for P_G6_. Moreover, the molecular function of the gene under the control of P_G1_ was determined to encode a high-affinity glucose transporter and named *GTH1*.

**Conclusions:**

A set of novel regulated promoters, enabling induction without methanol, was successfully identified by using DNA microarrays and shown to be suitable for high level expression of recombinant proteins in glucose-based protein production processes.

## Background

The methylotrophic yeast *Pichia pastoris* is widely used as a production platform for heterologous proteins. Latest developments in strain engineering for improved protein folding and secretion and glyco-engineering have recently been reviewed by Damasceno et al. [1].

Another important target for strain development is the promoter driving expression of the heterologous gene. A summary of the most important promoters of non-methylotrophic and methylotrophic yeasts is provided by Mattanovich et al. [2]. While production of recombinant proteins in *P. pastoris* has been successfully achieved under control of the constitutive glyceraldehyde-3-phosphate dehydrogenase promoter (P_GAP_), regulated promoters have several advantages: they enable initial biomass gain without product formation and allow tuning of the production process. Additionally, a potential impact of product accumulation on growth or viability of the cells can be prevented by decoupling growth from the production phase.

However, today’s available regulated promoters of *P. pastoris* have drawbacks. Many of them derive from methanol utilization pathway genes, which are generally repressed by glucose and/or ethanol and strongly induced by methanol. P_*AOX1*_ induces high-level expression of its encoded alcohol oxidase 1, which catalyzes the oxidation of methanol to formaldehyde [3]. Its weaker homolog P_*AOX2*_ has been used for protein production as well [4]. Another strong promoter of this pathway is P_*FLD1*_, its gene formaldehyde dehydrogenase is either induced by methylamine or methanol [5]. The promoter of dihydroxyacetone synthase, P_*DAS*_, was reported to be similarly regulated and even stronger than P_*AOX1*_[3], however it is not commonly used for protein production. Methanol and methylamine are both highly flammable and hazardous to health, so safety precautions are required for their industrial use. In addition to that, methanol consumption is technically disadvantageous because it causes high heat evolution and an increased oxygen demand during the fed batch phase [6].

The P_*ICL1*_ promoter controls the expression of isocitrate lyase and is regulated by the carbon source used for cell growth. No detectable promoter activity is present when cells are growing on glucose, while it gets turned on when cells are stationary or growing on ethanol [7]. Hence, this promoter might be an alternative for some applications, but its regulatory properties are poor. *PHO89* is a regulated sodium phosphate symporter and its promoter was investigated and shown to produce reasonable amounts of protein [8]. Cells must be phosphate-limited for the full activation of P_*PHO89*_, and an increase in product titer was even shown in phosphate-limited stationary phase. Additionally, an impact on growth was reported and reduced cellular fitness can be assumed in these conditions.

On the other hand, constitutive promoters might be advantageous for the over-expression of genes or to co-express helper factors and marker genes. The widely-used P_GAP_ controls the expression of glyceraldehyde-3-phosphate dehydrogenase at a high basal level [9]. Its productivity can be influenced by controlling the growth rate at the optimal activity of P_GAP_[10], and by a decrease of available O_2_ levels [11]. The promoter of the translational elongation factor EF-1 alpha gene, P_*TEF1*_, was analyzed and showed a tighter growth-associated regulation than P_GAP_[12].

A promoter library of P_*AOX1*_ was generated, leading to a few variants that were slightly stronger than wild type P_*AOX1*_, and a number of variants with altered regulatory properties, some of them being active without methanol [13]. Another library approach was done for P_GAP_ by mutation and clones expressing yeast-enhanced green fluorescent protein (yEGFP) under the control of obtained variants produced 8 to 218% of fluorescence intensity compared to the wild type promoter [14]. Potential promoter libraries can also be deduced from microarray data and rational considerations. Focussing on highly transcribed genes in general, 15 promoters were selected for characterization and the promoter of the thiamine biosynthesis gene P_*THI11*_, which is regulated by the availability of thiamine in the growth medium, was discovered [15].

As described above, the number of strong promoters with advantageous properties for protein production is limited in *P. pastoris*. This work was designated to identify novel promoters with both, high expression and an optimal regulation in production process conditions. Equally important, the addition of inducers was to be avoided, because their use is often associated with extra costs and safety precautions in large scale fermentation processes.

A typical production process under the control of P_GAP_ uses glycerol in the batch phase, and a constant glucose fed batch for 100 hours to reach more than 100 g L^-1^ cell dry weight [16]. In order to identify potential inducible promoters in the course of this process, we used DNA microarray analysis to compare gene expression patterns of glycerol-excess (=batch growth phase) and glucose-limited (=fed batch production phase) conditions. The expression capacity of selected promoter targets was characterized with model proteins and verified in fed batch processes.

## Results and discussion

### Identification of novel promoters with desired induction properties

A typical *P. pastoris* protein production process avoiding methanol induction starts with a glycerol batch (surplus of carbon source) which is followed by a glucose fed batch (limit of carbon source) [10]. DNA microarrays were used to analyze gene expression patterns and to identify potential promoters for this cultivation strategy. In order to eliminate growth rate related effects, glucose-limited conditions were analyzed in chemostat cultivation where the growth rate, similar to that in the batch phase, was fixed by controlling the dilution rate at 0.1 h^-1^.

**Figure 1:**
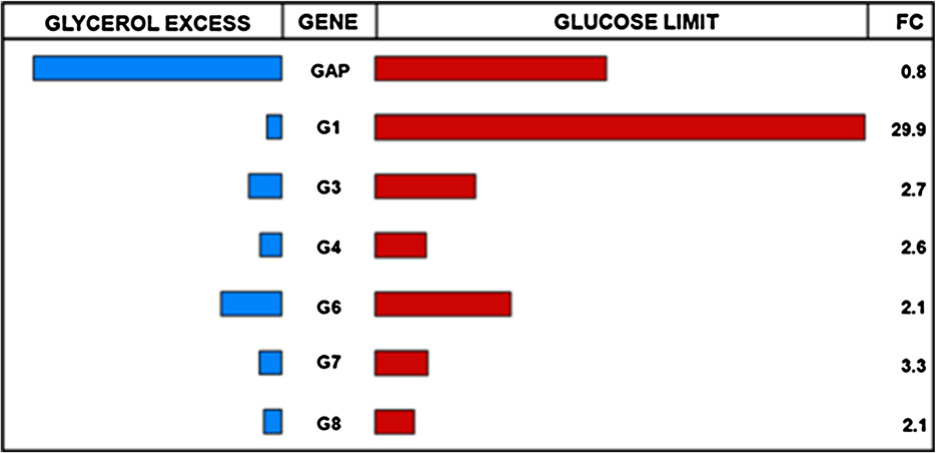
**Microarray data (red channel) of identified target genes in comparison to GAP.** Bars represent relative expression levels in glycerol excess (batch phase, blue bars on the left side) and in glucose limit (chemostat cultivation, red bars on the right side). Numbers in the right column represent the fold change of signal intensity between glucose limit and glycerol excess conditions.

The microarray data was mined for genes with both, high difference in expression level between repressed and induced state (fold change) as well as high signal intensity in the induced state to identify potent promoters for inducible high-level protein production in *P. pastoris*. Six potential promoters (abbreviated as P_G1_, P_G3_, P_G4_, P_G6_, P_G7_ and P_G8_, see Figure 1 and Table 1) were considered for further characterization.

**Table 1:** Identified promoter candidates

**Promoter**	**Gene**	***P. pastoris *gene identifier (strain GS115)**
P_GAP_	GAP	PAS_chr2-1_0437
P_G1_	G1	PAS_chr1-3_0011
P_G3_	G3	PAS_chr4_0550
P_G4_	G4	PAS_chr4_0043
P_G6_	G6	PAS_chr2-1_0853
P_G7_	G7	PAS_chr1-4_0570
P_G8_	G8	PAS_chr1-3_0165

### Verification of promoter strength and regulation

At first, the strength and regulation of the novel promoters were assayed with the intracellular reporter protein eGFP in small scale screening cultures. Both, repressive conditions in pre-culture (glycerol excess) and induced ones during main culture (glucose limit) were analyzed during the screening. In order to simulate fed batch like conditions in screenings, we had to adapt the screening strategy. Instead of usual feedings with certain amounts of glucose which lead to repeated batch phases, we used slow glucose releasing polymer particles (12mm feed beads, Kuhner, CH), liberating glucose at a non-linear rate of 1.63 ∙ t^0.74^ mg per disc (t = time [h]), which equals to 28.6 mg per disc after 48 hours.

**Figure 2:**
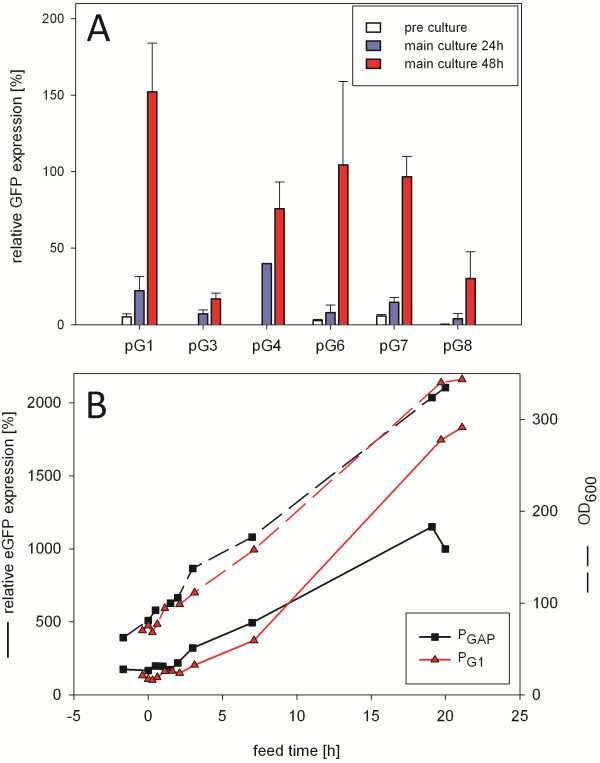
**Expression of eGFP under control of the novel promoters P**_**G1**_**, P**_**G3**_**, P**_**G4**_**, P**_**G6**_**, P**_**G7 **_**and P**_**G8**_**. (A)** Specific eGFP fluorescence in shake flask screenings related to P_GAP_ and to eGFP gene copy number. **(B)** Fed batch cultivations of single gene copy clones expressing eGFP under the control of P_GAP_ and P_G1_. Relative eGFP expression (solid lines) and OD_600_ (dashed lines) are shown over the feed time.

As shown in Figure 2, P_G1_ and P_G6_ had superior properties in terms of both, regulation and induction strength. In order to visualize gene dosage effects, genomic DNA of several clones was isolated and analyzed by real-time PCR to determine the gene copy number (GCN) of eGFP. Compared to a P_GAP_ clone with one gene copy, the specific fluorescence of P_G1_ and P_G6_ controlled expression of eGFP (normalized to GCN) were induced from almost zero in batch phase to about 150% and 100% after 48 h screening culture, respectively. The other promoters P_G3_*,* P_G4_, P_G7_, and P_G8_ still showed a good regulation and induction strength suitable for inducible protein expression, with expression strengths spanning a spectrum of about 20% to 120% relative to P_GAP_ (Figure 2A). The next step was to investigate the induction behaviour of the novel promoters in more detail.

### Analysis of the glucose dependent regulation

The induction behaviour of the novel promoters was characterized in screenings with eGFP producing clones in YP media containing different amounts of glucose (ranging from 20 to 0.002 g L^-1^). The cells were cultivated for 5–6 hours and eGFP expression was analyzed by flow cytometry.

**Figure 3:**
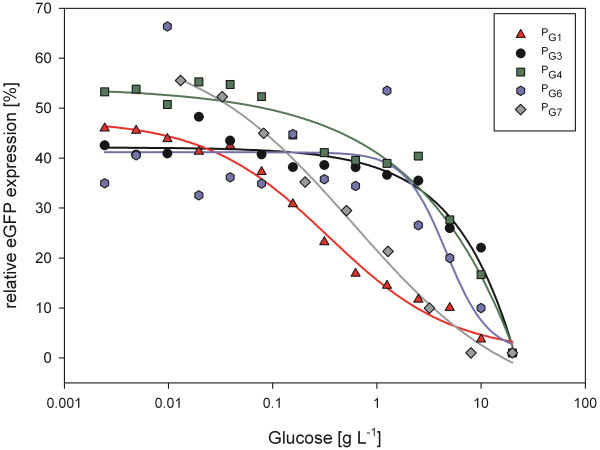
**Induction behaviour of the novel promoters.** Specific eGFP fluorescence of clones expressing eGFP under the control of P_G1_, P_G3_, P_G4_, P_G6_ and P_G7_ in media containing different amounts of glucose. Data is related to P_GAP_, normalized to 1.0 at the highest glucose concentration of 20 g L^-1^ and plotted against the logarithmic glucose concentration (trend line calculation: four parameter logistic curve). The glucose concentration given on the x-axis refers to the glucose set point at the beginning of the cultivation (serial dilutions ranging from 20 to 0.002 g L^-1^). This screening setup and data processing points out relative promoter activities, thereby showing the kinetics of induction, but does not allow comparison of promoter strength.

Promoters P_G1_ and P_G7_ showed a flat induction course leading to full activity only with less than 0.05 g L^-1^ glucose. That is clearly different to P_G3_, P_G4_ and P_G6_´s steeper regulation pattern which reach their top activity already at around 4 g L^-1^ glucose (Figure 3). In other words, P_G1_ is not only the strongest but also most tightly repressed by glucose among the promoters tested here.

Based on these regulatory features we intended to characterize the functions of the genes under control of the P_G_ promoters. At the time of their identification, no or only putative functions were assigned to the underlying genes aside from G1. Therefore, we used NCBI Conserved Domain search to analyze the protein sequences in order to identify putative gene functions.

The gene under the control of P_G1_ was previously functionally clustered with *K. lactis* high-affinity glucose transporter *HGT1*[17]. It contains two major facilitator superfamily domains, same as the G7 gene, which is therefore assumed to be a glucose transporter too. For *Saccharomyces cerevisiae*, it was reported that hexose transporters underlie complex regulation patterns and are expressed in dependence of glucose concentration [18]. The regulation pattern exhibited by the promoters P_G3_, P_G4_, P_G6_ and P_G8_ might be associated with a role in central metabolism. An AKR (aldo keto reductase) domain was found in the gene expressed under P_G3_. The genes controlled by P_G4_ and P_G6_ are both putative aldehyde dehydrogenases, predicted to be localized in the cytosol and in the mitochondria, respectively. The Gti1/Pac2 family domain found in G8 plays a role in gluconate uptake upon glucose starvation and in sexual development in *Schizosaccharomyces pombe*.

We could show the need of an explicit glucose limit for full activity of the novel promoters, which is most pronounced for P_G1_. This demonstrates that the microarray data-based promoter selection is excellently suited to select for promoters with features relevant for bioprocesses, and secondly indicates the novel promoter´s advantages in fed batch fermentation.

To prove this statement, the application of the strongest and most promising promoter P_G1_ was tested in a fed batch fermentation where truly glucose-limited conditions are present [10]. A single gene copy clone expressing eGFP was chosen for comparison to an equivalent single gene copy clone of eGFP under the control of P_GAP_. Thereby we could show that the P_G1_ promoter remains repressed during the batch phase and that its induction during fed batch clearly exceeds the strength of P_GAP_. Relative eGFP expression (fluorescence related to the culture volume) and OD_600_ over the feed time are shown in Figure 2B.

### Expression of secreted human serum albumin

**Figure 4:**
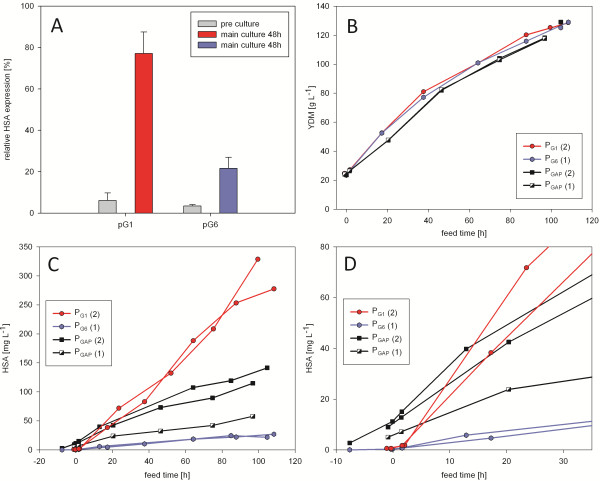
**Expression of secreted HSA using the novel promoters P**_**G1 **_**and P**_**G6 **_**in shake flask and fed batch cultivations. (A)** HSA expression in shake flask screenings related to P_GAP_ and to the gene copy number. **(B)** Dry cell weight and **(C)** HSA titer in fed batch cultivations of double and single gene copy clones expressing under the control of P_G1_ (circle, two copies), P_G6_ (diamond, one copy) and P_GAP_ (black square, two copies and black-and-white, one copy). **(D)** Detail of (**C**) showing late batch and early fed batch phase, highlighting the different regulation properties of the promoters. Except for the single gene copy P_GAP_ clone, all fermentations were performed in duplicates.

P_G1_ and P_G6_ were further selected to assay the production of a secreted protein under their control. Human serum albumin (HSA) is efficiently produced in *P. pastoris*. Therefore it can be regarded as an industrially relevant secreted reporter protein. Its expression under the control of P_G1_, P_G6_ and P_GAP_ was screened in shake flasks with glucose-limited conditions (through the use of feed beads) in the main culture. During the glycerol (batch) pre-culture, both P_G1_ and P_G6_ promoters remained well repressed. As seen before, during main culture expression under P_G1_ was stronger than under P_G6_ and, in relation to gene copy number, reached around 77% of the biomass specific HSA yield of cells expressing under P_GAP_, while P_G6_ produced about 22% of P_GAP_ (Figure 4A). However, the novel promoters might not show their full potential in shake flask screenings, since these conditions are not strictly glucose limited during the entire production phase.

To exploit the full potential of the novel promoters, glucose limited fed batch cultivations were performed. Based on the screening results, one clone each expressing sufficient amounts of HSA under the control of P_G1_ and P_G6_ was selected. Those clones, harbouring two and one gene copies respectively, were compared with their respective gene copy equivalent P_GAP_ clones (Figure 4B, 4C and 4D). Dry cell weight (DCW) and HSA titers are summarized in Table 2. HSA titers of P_GAP_ clones correlate with their respective gene copy number. Again, the P_G1_ clone showed superior properties - it clearly outperformed the P_GAP_ clone with the same gene copy number and produced about 230% of the biomass specific product yield compared to P_GAP_. P_G6_ produced about 39% of the biomass specific HSA yield of its gene copy equivalent P_GAP_ clone. Besides, PGAP-driven expression was already active in the batch phase, and more than 5% of the final HSA amount was already present at the batch end for both clones expressing under its control. While this is not an issue in case of HSA, it is a clear disadvantage compared to inducible promoters such as P_G1_ in case of toxic or difficult to express products. Figure 4C shows HSA titer over the feed time, and the unique repression/induction efficiency of P_G1_ is clearly pointed out in the first hours (Figure 4D). Both, P_G1_ and P_G6_ showed good repression in the batch phase and induction by the glucose limited feed.

**Table 2:** **Summary of fed batch cultivations of*P. pastoris*expressing HSA under the control of P**_**G1**_**, P**_**G6**_**and P**_**GAP**_

		**Batch end**			**Fed batch end**			
**Promoter**	**GCN**	**DCW** **[g L**^**-1**^**]**	**HSA** **[mg L**^**-1**^**]**	**HSA/DCW** **[mg g**^**-1**^**]**	**DCW** **[g L**^**-1**^**]**	**HSA** **[mg L**^**-1**^**]**	**HSA/DCW** **[mg g**^**-1**^**]**	**% HSA/DCW** **of P**_**GAP**_
P_G1*_	2	24.3	0.5	0.0	126.9	303.1	2.4	231.2
P_G6*_	1	23.9	0.3	0.0	127.1	24.3	0.2	38.9
P_GAP*_	2	23.8	10.1	0.4	123.7	128.2	1.0	
P_GAP_	1	24.2	5.0	0.2	117.7	57.8	0.5	

* performed in duplicates.

To verify that the novel promoters also exhibit their superior regulatory properties and expression capacity in industrially relevant conditions, we elevated HSA gene copy number by post-transformational vector amplification as described previously [19]. Thereby, we were able to produce more than 1 g L^-1^ HSA under the control of P_G1_ with a clone harbouring 11 gene copies, which corresponds to the 3.4-fold titer of its two copy clone (Table 3). Again, P_G1_ outperformed a comparable clone with the same gene copy number under the control of the P_GAP_ promoter, which produced 607 mg L^-1^ HSA in a similar fermentation [19]. High level HSA production was also achieved with an amplified clone expressing HSA (11 gene copies as well) under the control of the weaker P_G6_ promoter, which produced more than 720 mg L^-1^ HSA (Table 3). This titer is approximately 30-fold higher than the titer reached with the P_G6_ single copy clone (24 mg L^-1^), thus indicating that multiple copies of expression cassettes under control of a weaker promoter can also lead to high productivities. One possible explanation of this effect could be that the ratio of a repressing protein to promoter copy number and thus repressor binding sites is decreased in the amplified clones, therefore leading to higher transcription.

**Table 3:** **Summary of fed batch cultivations of GCN amplified HSA expressing clones under the control of P**_**G1**_**and P**_**G6**_

		**Batch end**			**Fed batch end**		
**Promoter**	**GCN**	**DCW** **[g L**^**-1**^**]**	**HSA** **[mg L**^**-1**^**]**	**HSA/DCW** **[mg g**^**-1**^**]**	**DCW** **[g L**^**-1**^**]**	**HSA** **[mg L**^**-1**^**]**	**HSA/DCW** **[mg g**^**-1**^**]**
P_G1_	11	18.9	0.2	0.0	114.0	1060.8	9.3
P_G6*_	11	22.5	0.3	0.0	110.8	728.7	6.6

* performed in duplicates.

Additionally, the porcine enzyme carboxypeptidase B (CpB) that is used for human insulin production, and an antibody Fab fragment were produced under the control of P_G1_ and P_G6_ respectively, and exceeded the production levels compared to P_GAP_. Thereby we verified again the suitability of these promoters in standard glucose based production processes.

### P_G1_ activity depends on specific growth rate

**Figure 5:**
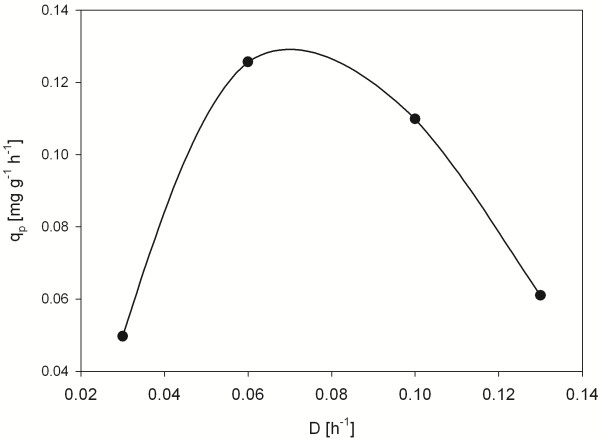
**Correlation of specific productivity to specific growth rate using P**_**G1**_**. ** Specific product formation rate (q_p_) observed in chemostat cultivation at different dilution rates of a clone expressing HSA under the control of P_G1_ as well as the respective trend curve (spline curve).

We elucidated the expression activity of P_G1_ at different growth rates using an HSA clone with two gene copies under its control. It was cultivated in chemostat with different dilution rates and the highest specific product formation was found at a growth rate of about 0.07 h^-1^ (Figure 5). This clearly differs to the profile obtained with P_GAP_ in [10], where the highest specific product formation was obtained only at higher growth rates. Growth rate dependency may be utilized to optimize space-time yield or other parameters in the production processes [10].

### Knock out of G1

Furthermore, we decided to clarify the function of the gene PAS_chr1-3_0011, which underlies the control of the promoter P_G1_. It contains 12 transmembrane domains (predicted by TMHMM Server v. 2.0), two Major Facilitator Superfamily (MFS) and other transporter domains. Based on the sequence homology to the *K. lactis* high- affinity glucose transporter *HGT1*, the gene controlled by P_G1_ was expected to have a function in glucose transport [17], [20]. Strong activity of its promoter at very low glucose concentrations further strengthened this assumption. For further verification, the gene was disrupted using the split marker cassette technique (primers given in Additional file 1: Table S1) as described by Heiss et al. [21]. Similar as described by Jørgensen and his colleagues [22], we compared the glucose uptake of the wild type and a G1 knock out clone in glucose-limited chemostat cultivations at different growth rates. The glucose saturation constants were calculated from the residual glucose concentrations (Table 4) and a K_S_ of 9.7, 23.1 and 69.3 μM was obtained for three different dilution rates (μ=0.14, 0.1 and 0.05 h^-1^) for the wild type. Changing K_S_ values are observed for the whole cell in different conditions, which is due to the differential regulation of its several transporters. A reduced capacity of glucose uptake at low specific growth rates has been reported before [22]. The G1 knock out clone appeared to have much higher saturation constants of 90.4, 99.0 and 207.8 μM at the same dilution rates, which was also described for the high-affinity glucose transporter disruption in *Aspergillus niger*[22]. The knock out clone does not display the low K_S_ values of the wild type, so that the gene PAS_chr1-3_0011 was clearly identified as a high-affinity glucose transporter. As the short name *HGT1* is used as an alias for a peptide transporter in *S. cerevisiae* we suggest the short name *GTH1* (glucose transporter with high affinity) for this *P. pastoris* gene.

**Table 4:** Glucose substrate saturation constants of a wildtype and a G1 knock out clone

	**G1 k. o., μ**_**max**_**= 0.18 h**^**-1**^			**Wildtype, μ**_**max**_**= 0.18 h**^**-1**^		
D [h^-1^]	S [μM]	X [g L^-1^]	K_S_ [μM]	S [μM]	X [g L^-1^]	K_S_ [μM]
0.14	316.4	29.5	90.4	33.9	30.5	9.7
0.10	123.8	31.3	99.0	28.9	32.1	23.1
0.05	79.9	31.3	207.8	26.6	30.8	69.3

Residual glucose (S), dry cell weight (X) and substrate saturation constant (K_S_) of a wild type and a G1 knock out (k. o.) clone at different dilution rates in chemostat cultivations.

## Conclusions

Efficient regulated promoters cannot necessarily be found by classical batch screening approaches. Simulating production conditions in lab scale and searching the promoter space offers a new target oriented approach. We could show here that the cultivation of *P. pastoris* in conditions where repression or induction are desired, followed by the analysis of transcript levels with DNA microarrays offers a potent opportunity to find new, strong and regulated promoters.

Six novel promoters were identified and further characterized. All of them are activated by carbon source depletion. The new promoters provide a tool box for expression of recombinant genes and are thus well suitable for protein production processes. P_G1_ had the most favourable repression kinetics and exceeded the expression levels of the well-established constitutive GAP promoter in glucose limited fed batch cultures by more than twofold. The molecular function of the gene under its control was identified as high-affinity glucose transporter and named *GTH1*.

## Materials and methods

### Strains and cultivation

*Escherichia coli* DH10B (Invitrogen) was used for subcloning. It was routinely cultivated in petri dishes or shake flasks using LB media supplemented with 25 μg mL^-1^ Zeocin. A wild type *Pichia pastoris* strain CBS2612 which can grow on minimal media supplemented with biotin, was used for protein expression in this work.

The main culture for screenings was either done with YP or BM media and glucose feed beads (12 mm, Kuhner, CH) which provided the carbon source.

YP media contained 20 g L^-1^ peptone and 10 g L^-1^ yeast extract, which can be supplemented with 12.6 g glycerol or 20 g glucose to obtain YPG and YPD, respectively. For cultivation on plates, 5 g L^-1^ agar-agar was added to the liquid medium. BM media was based on YP, supplemented with 13.4 g L^-1^ yeast nitrogen base (Cat.No. 291940, Becton Dickinson, FR) with ammonium sulfate, 0.4 mg L^-1^ biotin and 100 mM potassium phosphate buffer pH 6.0.

### Identification of novel inducible promoters

a) Bioreactor cultivations

Fermentations for the identification of promoter candidates were done in 3.5 L working volume bioreactors (Minifors, Infors, CH) in three biological replicates. Cells were grown for about 24 h in batch on glycerol medium, followed by an exponential feed phase on glycerol fed batch medium calculated as described by Resina et al. [23] with a specific growth rate of μ= 0.1 h^-1^ and a substrate yield coefficient of Y_X/S_ of 0.5 g g^-1^. Sequentially, chemostat cultivation (D = μ = 0.1 h^-1^) with high density glucose medium was performed.

Glycerol batch medium contained per liter: 2 g citric acid monohydrate, 39.2 g glycerol, 20.8 g NH_4_H_2_PO_4_, 0.5 g MgSO_4_∙ 7H_2_O, 1.6 g KCl, 0.022 g CaCl_2_∙ 2H_2_O, 0.8 mg biotin and 4.6 mL PTM1 trace salts stock solution. HCl was added to set the pH to 5.0.

Glycerol fed-batch medium contained per liter: 632 g glycerol, 8 g MgSO_4_∙ 7H_2_O, 22 g KCl, and 0.058 g CaCl_2_∙ 2H_2_O.

High-density chemostat medium contained per liter: 2 g citric acid monohydrate, 99.42 g glucose monohydrate, 22 g NH_4_H_2_PO_4_, 1.3 g MgSO_4_∙ 7H_2_O, 3.4 g KCl, 0.02 g CaCl_2_∙ 2H_2_O, 0.4 mg biotin and 3.2 mL PTM1 trace salts stock solution. HCl was added to set the pH to 5.0.

PTM_1_ trace salts stock solution contained per liter: 6.0 g CuSO_4_∙ 5H_2_O, 0.08 g NaI, 3.36 g MnSO_4_∙ H_2_O, 0.2 g Na_2_MoO_4_∙ 2H_2_O, 0.02 g H_3_BO_3_, 0.82 g CoCl_2_, 20.0 g ZnCl_2_, 65.0 g FeSO_4_∙ 7H_2_O, 0.2 g biotin and 5.0 mL H_2_SO_4_ (95%-98%).

b) Microarray hybridization

RNA purification and sample preparation as well as microarray hybridization (in-house designed *P. pastoris* specific oligonucleotide arrays, AMAD-ID: 034821, 8x15K custom arrays, Agilent) and data analysis were done as described by Graf et al. [24].

### Characterization of promoter strength and regulation

a) Cloning

Cloning and transformation was done using the in-house vector pPuzzle [15], which contains a Zeocin resistance cassette for selection in both *E. coli* and yeast, an expression cassette for the gene of interest (GOI) consisting of a multiple cloning site and the *S. cerevisiae* CYC1 transcription terminator, and a locus for integration into the *P. pastoris* genome (3´ AOX1 region or rDNA locus). Promoter sequences (up to 1000 bps upstream of the start codon of their respective genes) were PCR-amplified from *P. pastoris* genomic DNA (primer sequences see Additional file 1: Table S1). The promoters were ligated into pPuzzle in front of the start codons of the model proteins, using the ApaI and the SbfI restriction sites of the multiple cloning site of the vector. Vectors expressing the respective model protein under control of P_GAP_ were used as controls throughout the study. For the expression of heterodimeric HyHEL antibody Fab fragment (HyHEL Fab), the expression cassettes of light chain and Fab heavy chain (each under control of P_G1_) were combined into one vector (using the strategy described in [27]).

HSA was secreted by its native secretion leader, while for CpB and HyHEL Fab the *S. cerevisiae* alpha mating factor signal sequence was used. To avoid positional effects on reporter gene expression levels, genome integration of the expression plasmids was targeted to either the 3´flanking region of the *AOX1* gene or the ribosomal DNA locus (rDNA, for multicopy integration) of *P. pastoris,* respectively.

Plasmids were linearized within the genome integration region prior to electroporation (2 kV, 4 ms, GenePulser, BioRad) into electrocompetent *P. pastoris*. Multicopy integration of HSA expressing clones was done as described by Marx et al. [19] and selected at higher Zeocin concentrations (up to 1000 μg mL^-1^).

*P. pastoris* cells were first selected and cultivated in petri dishes on YPD agar and then inoculated in an YPG medium as pre-culture for screenings and fermentations. Antibiotic selection by Zeocin was applied on plates and in pre-culture at a concentration of 25 μg mL^-1^ or higher.

b) Expression screening

Expression of intracellular eGFP and the secreted proteins HSA, CpB and HyHEL Fab with the novel promoters in comparison to P_GAP_ was evaluated in shake flask screenings. All screenings were performed at 25°C and with shaking at 180 rpm. Single colonies were inoculated in YPG medium with selection pressure (Zeocin) for pre-culture. After approximately 24 hours, the pre-culture was used to inoculate the main culture with an optical density (OD_600_) of 0.1 (for eGFP) or 1 (for HSA, CpB and HyHEL Fab) in 10 mL YP or BM medium, respectively. Glucose feed beads (12 mm, Kuhner, CH) were used to generate glucose-limiting growth conditions. Expression of eGFP was measured at the end of pre-culture and at 24 and 48 hours of the main culture. Culture supernatant of clones expressing secreted protein was harvested from the pre-culture and after 48 hours and cell density was determined by measuring wet cell weight or OD_600_.

c) Comparative analysis of *P. pastoris* promoter activity

In order to analyze relative transcription strength of the P_G_ promoters at different glucose concentrations, a comparative promoter activity study using various glucose concentrations (ranging from 20 to 0.002 g L^-1^ glucose) was performed with eGFP expressing clones in 24-well plates (Cat. No. 7701–5110, Whatman, UK) covered with breath seal membranes (Cat. No. B-100, Excel Scientific, CA). Glucose concentrations of 20, 10, 5, 2.5, 1.25, 0.63, 0.31, 0.16, 0.08, 0.04, 0.02, 0.01, 0.005, 0.002 g L^-1^ were obtained by serial dilution in YP media, and represent the inital setpoints. The main culture was inoculated from YPG-Zeocin pre-culture with an OD_600_ of 0.01 and samples were taken after 5–6 hours and analyzed by flow cytometry.

d) Fed batch cultivation

All fed batch fermentations were done in 1.0 L working volume bioreactors (SR0700ODLS, DASGIP, DE). The dissolved oxygen was controlled at DO = 20% with the stirrer speed (400 – 1200 rpm). Aeration rate was 18 L h^-1^ air, the temperature was controlled at 25°C and the pH was controlled at 5.85 for HSA [25] or pH 5.0 for the other proteins [10] with addition of ammonium hydroxide (25%). To start the fermentation, 300 mL batch medium was sterile filtered into the fermenter and a *P. pastoris* clone was inoculated from an overnight pre-culture with a starting optical density (OD_600_) of 1. For the cultivation of clones expressing eGFP, the batch phase of approximately 25 h was followed by a fed batch phase with a feeding rate optimized according to [10]. HSA expressing strains were cultivated as described by Marx et al. [19], where the batch phase was followed by a constant feed of 2 g h^-1^ fed batch medium for 100 h, Carboxypeptidase B and HyHEL Fab expressing clones were cultivated similarly. Samples were taken during batch and fed batch phase, and analyzed for expression.

Glycerol batch and glucose fed batch media for eGFP, HyHEL Fab and Carboxypeptidase B expressing clones were exactly as described in [10], while for the production of HSA the media was described in [19].

e) Chemostat cultivation

A strain expressing HSA (2 GCN) under control of P_G1_ was tested for its growth rate dependent expression behaviour in chemostat at different dilution rates (D = 0.03, 0.06, 0.10, 0.13) [10].

For characterization of glucose uptake characteristics, the *P. pastoris* wild type strain and the strain deleted for PAS_chr1-3_0011 were cultivated in glucose limited chemostats at D = 0.05, 0.10, and 0.14 h^-1^. Samples were taken rapidly as described below.

### Analytical methods

a) Copy number determination with real-time PCR

Genomic DNA was isolated using the DNeasy Blood&Tissue Kit (Cat. No. 69504, Quiagen, DE). Gene copy numbers were determined with quantitative PCR using the SensiMix SYBR Kit (QT605-05, Bioline reagents, UK). The primers (supplementary Additional file 1: Table S1) and sample were mixed with the SensiMix and applied for real time analysis in a real-time PCR cycler (Rotor Gene, Qiagen, DE). All samples were analyzed in tri- or quadruplicates. Data analysis was performed with the two standard curve method of the Rotor Gene software. The actin gene *ACT1* was used as calibrator.

b) Determination of protein expression levels

A plate reader (Infinite 200, Tecan, CH) was used to determine eGFP fluorescence in fermentation samples. Therefore, samples were diluted to an OD_600_ of 5 and fluorescence intensity was then related to the culture volume.

Expression of eGFP in screenings was analyzed by flow cytometry as described before [15]. Specific eGFP fluorescence referred to in this study is the fluorescence intensity related to the cell volume for each data point as described by Hohenblum et al. [26]. Then the geometric mean of the population´s specific fluorescence was normalized by subtracting background signal (of non-producing *P. pastoris* wild type cells) and related to expression under the control of P_GAP_.

For quantification of HSA in shake flask and fermentation supernatants, the Human Albumin ELISA Quantitation Set (Cat. No. E80-129, Bethyl Laboratories, TX) was used. The HSA standard was applied with a starting concentration of 400 ng mL^−1^. Dilution-, Blocking- and Washing buffer were based on TBS (50 mM Tris–HCl, 140 mM NaCl, pH 8.0) and completed with BSA (1% (w/v)) and/or Tween20 (0.05% (v/v)) accordingly.

HyHEL Fab was determined with ELISA as described previously [27].

CpB was quantified using an enzymatic assay based on the cleavage of hippuryl-L-arginine (Cat. No. H2508, Sigma, MO). Generation of hippuric acid was monitored at its absorbance maximum of 254 nm. Prior to the measurement, the samples were desalted with Zeba Spin columns (Thermo Fisher Scientific, IL) and activated with trypsin (Cat. No. T8345, Sigma, MO).

c) Determination of residual glucose

The D-Glucose Assay - GOPOD-Format (Megazymes, IE) was used to determine residual glucose of chemostat samples. Supernatant sampling was done by pumping culture broth out of the bioreactor by producing an overpressure, and its direct sterile filtration using a vacuum filter unit (Cat. No. 5141178, Whatman, UK). Glucose-limited cultivations usually go along with very low residual glucose concentrations in the supernatant, so the manufacturer’s protocol was adapted for glucose concentrations from 10 to 100 mg L^-1^. Briefly, the ratio of reaction buffer to sample was changed from 30:1 to 3:1.

## Competing interests

The authors declare that they have no competing interests.

## Authors’ contributions

RP performed the experimental work, data analysis, contributed to study design and drafted the manuscript. MM contributed to study design and planning of bioreactor cultivations. JK and JW conceived and provided industrially relevant screening conditions. CK supported data interpretation and planning of promoter characterization. BG planned and supervised the experimental work, and contributed to data analysis and drafting the manuscript. DM coordinated the project and contributed to drafting the manuscript. BG, MM and DM conceived of the study. All authors read and approved the final manuscript.

## Supplementary Material

Additional file 1: Table S1 Primer Sequences. Sequences of oligonucleotides used for amplification of promoters, determination of gene copy numbers of the model protein expression cassettes, and generation of G1 disruption cassette (including verification of positive knock-outs). Click here for file
