# Supplementary material for: Induction without methanol: novel regulated promoters enable high-level expression in Pichia pastoris
Source: Microb Cell Fact. 2013 Jan 24;12:5. doi: 10.1186/1475-2859-12-5 (PMC3615954; doi:10.1186/1475-2859-12-5)
Supplement: Additional file 1: Table S1 — Primer Sequences. Sequences of oligonucleotides used for amplification of promoters, determination of gene copy numbers of the model protein expression cassettes, and generation of G1 disruption cassette (including verification of positive knock-outs). [file 1475-2859-12-5-S1.pdf]

Supplementary Table S1 Primer Sequences

Primer name	Sequence
Primers for the amplification of P. pastoris promoters	
P_{GAP}_fw	5'-AACCGGGCCCAGATCTTTTTTGTAGAAATGT-3'
P_{GAP}_back	5'-CATGGCCTGCAGGTGATAGTTGTTCAATTGATTGAAATAGGGACAAAT-3'
P_{G1}_fw	5´-GATA*GGGCCC*CAAACATTTGCTCCCCCTAGTCTC-3´
P_{G1}_back	5´-GATA*CCTGCAGG*AAGGGTGGAATTTTAAGGATCTTTTAT-3´
P_{G2}_fw	5´-GATA*GGGCCC*AATACCTTCGGTAACATATT-3´
P_{G2}_back	5´-GATA*CCTGCAGG*GTTTACTCGTATATAGAATGGTAGGTTCAAT-3´
P_{G3}_fw	5´-GATA*GGGCCC*CAGCAATCCAGTAACCTTTTCTGAAT-3´
P_{G3}_back	5´-GATA*CCTGCAGG*TTGAGTTCAATAAATTGTCCGGGA-3´
P_{G4}_fw	5´-GATA*GGGCCC*TGGACTGTTCAATTTGAAGTCGATG-3´
P_{G4}_back	5´-GATA*CCTGCAGG*GGATAAAGGTAAGGGAAAAAAGCAA-3´
P_{G5}_fw	5´-GATA*GGGCCC*CATTCAGTGGACTGCTAAATGCTTTA-3´
P_{G5}_back	5´-GATA*CCTGCAGG*TTTTCAAAAAATGGCCCACACA-3´
P_{G6}_fw	5´-GATA*GGGCCC*AGACCAGCAGTTTAACTACGCAAATC-3´
P_{G6}_back	5´-GATA*CCTGCAGG*CTTTTCTTTGGGCAAGGAAAAATC-3´
Primers for real-time PCR	
PpACT1_Up	5´-CCTGAGGCTTTGTTCCACCCATCT-3´
PpACT1_Low	5´-GGAACATAGTAGTACCACCGGACATAACGA-3´
PpeGFP_Up	5´-TCGCCGACCACTACCAGCAGAA-3´
PpeGFP_Low	5´-ACCATGTGATCGCGCTTCTCGTT-3´
PpHSA_Up	5´-AAACCTAGGAAAAGTGGGCAGCAAATGT-3´
PpHSA_Low	5´-ACTCTGTCACTTACTGGCGTTTTCTCATG-3´
HC_up	5´-TACTGCTGCTTTGGGTTGTTTGGT-3´
HC_low	5´-AAGGGACAGTAACAACAGAGGACA-3´
LC_up	5´-GATGAACAATTGAAGTCTGGTAC-3´
LC_low	5´-GAGTAACTTCACAAGCGTAAACC-3´
CpB_up	5´-GCTGGAGGTTCTGATGATTGGGCTTACGAC-3´
CpB_low	5´-TGAATCTGGGACTCTGGCAAAATGAAACC-3´
Primers for the generation of the G1 knock out casettes	
G1KO A bw	5´-GGAGTAGAAACATTTTGAAGCTATGGTCCAGACCACCGTATTATCCGTAAT-3´
G1KO B fw	5´-ATTACGGATAATACGGTGGTCTGGACCATAGCTTCAAAATGTTTCTACTCC-3´
G1KO B bw	5´-CTGCTCGCCGATCTCGGTC-3´
G1KO C fw	5´-TGACCAGTGCCGTTCCGG-3´
G1KO C bw	5´-ACGGTTGAAGTAGTTTCTGTAATGATCC AATTAAAGCCTTCGAGCGTCCC -3´
G1KO D fw	5´-GGGACGCTCGAAGGCTTTAATT GGATCATTACAGAAACTACTTCAACCGT -3´
G1KO D bw	5´-CGAATAGATAGGAACAGGCAATAACAC -3´
